# Epithelial conjunctival neoplasias – the importance of an early diagnosis and optimal treatment

**DOI:** 10.3205/oc000157

**Published:** 2020-08-06

**Authors:** Laura Tabuenca Del Barrio, Marcos Mozo Cuadrado, Luiz Miguel Nova Camacho, Alicia Zubicoa Enériz, Miren Dolores Aranguren Laflin, Araceli Alcaine Soler

**Affiliations:** 1 Ophthalmology, Complejo Hospitalario de Navarra, Pamplona, Navarra, Spain; 2 Pathology, Complejo Hospitalario de Navarra, Pamplona, Navarra, Spain

**Keywords:** squamous cell carcinoma, conjunctival malignancies, CIN, exenteration, excisional biopsy, radiotherapy

## Abstract

**Objective:** To emphasize the importance of an early diagnosis and an adequate treatment in conjunctival tumors.

**Methods:** We present two clinical cases and compare the course of each case: one of conjunctival intraepithelial neoplasia (CIN) which took a positive course, and a fatal case of squamous cell carcinoma (SCC) with intraocular and orbital extension.

**Results:** Epithelial conjunctival malignancies are one of the most prevalent ocular surface tumors. Among these, CIN are the most common. CIN have an excellent prognosis, given adequate treatment. However, when the diagnosis of CIN is late, the epithelial basement membrane will be affected, resulting in SCC. SCC may have poorer results due to its capacity to infiltrate near tissues and create distant metastasis.

**Conclusion:** It is not common today to treat patients with orbital extension of SCC; however, it is crucial to note the importance of an early diagnosis of conjunctival malignancies. An early diagnosis is essential to prevent the transformation to other life-threatening types.

## Introduction

Conjunctival tumors are one of the most common ocular surface malignancies [1], [2]. Their most frequent cell origin is located on epithelial and melanocytic cells. Since in most cases it is very difficult to differentiate between premalignant and malignant lesions, a biopsy is necessary to determine their nature [1].

Epithelial neoplasms can be divided into

1. carcinoma in situ/conjunctival intraepithelial neoplasia (CIN), which exclusively affect the epithelium;
2. invasive squamous cell carcinomas (SCC), when the epithelial basement membrane is damaged and neoplastic cells are present in the corneal stroma [3].

We report two clinical cases of conjunctival neoplasm. The first one exhibits a patient affected by CIN with an early diagnosis and effective treatment based on topical chemotherapy and surgical excision. In contrast, the second case shows a patient with SCC with an aggressive clinical course and intraocular and orbital extension.

The fact that the cell origin was the same in both cases shows that an adequate diagnosis and optimal treatment are required to control the progression of these types of tumors. Intraocular and orbital extension is infrequent but commonly fatal.

## Case descriptions

### Case 1

**Figure 1:**
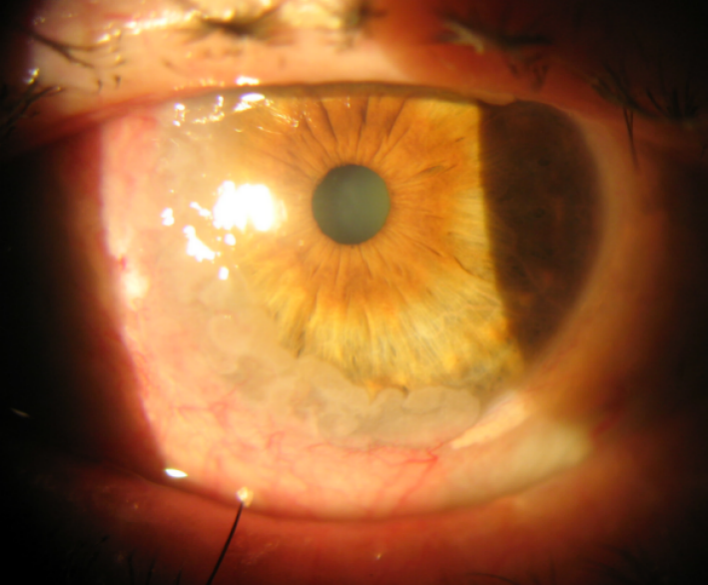
Slit-lamp examination; nasal limbal gelatinous mass with inferior corneal infiltration

**Figure 2:**
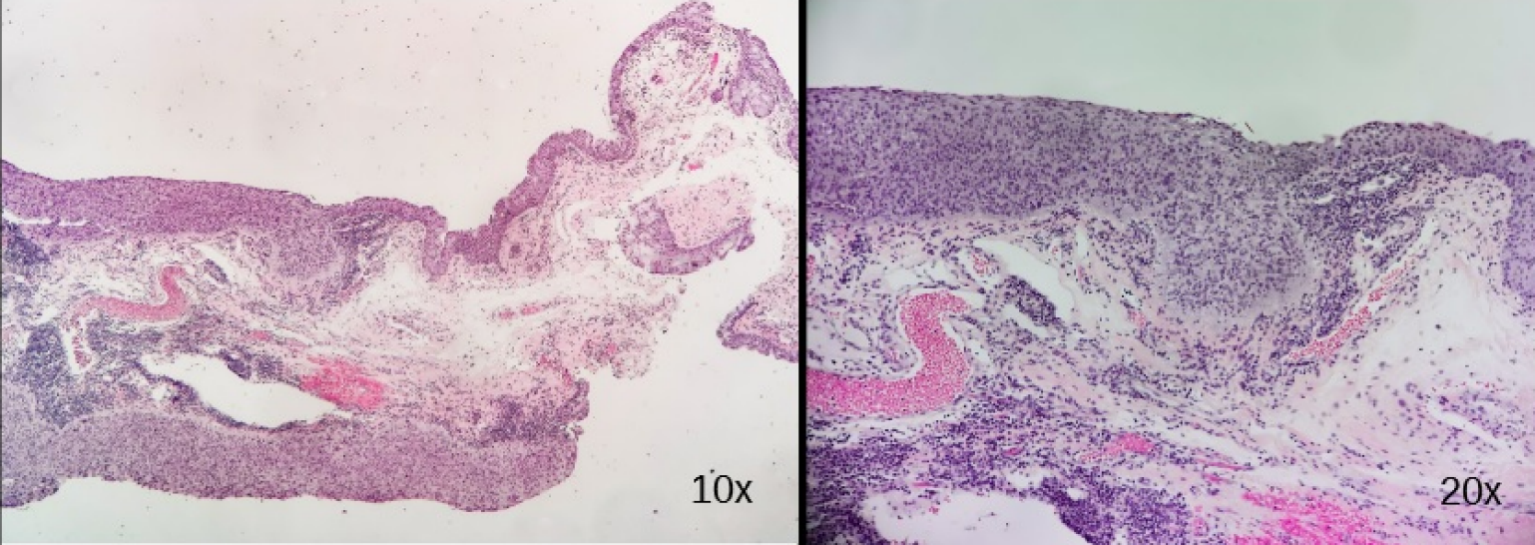
CIN was confirmed by histopathological study with free margins; normal conjunctival epithelium at the top of the biopsy and dysplastic cells at the bottom in image 10x; dysplastic epithelium with high mitosis index in image 20x

**Figure 3:**
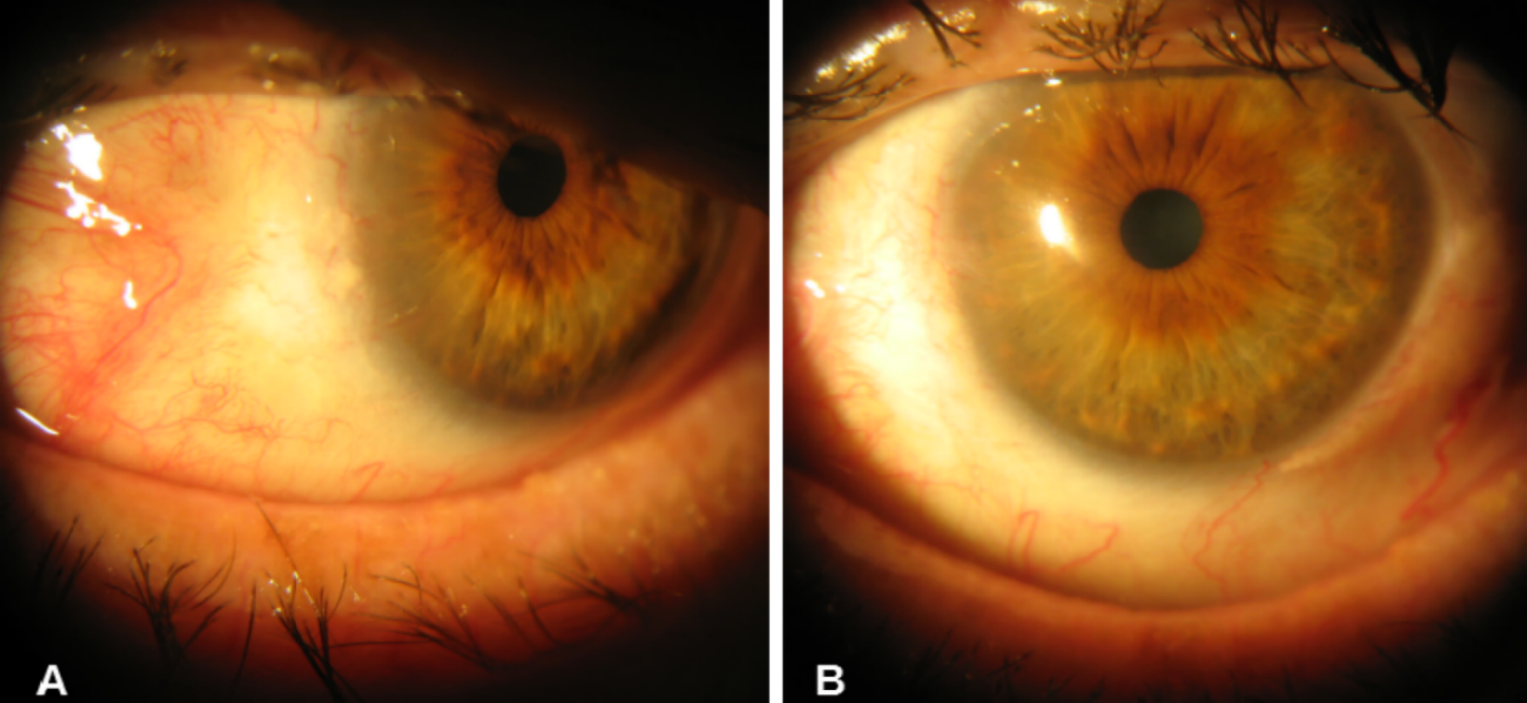
A) One month later, the lesion had decreased. B) At the third month, CIN had disappeared.

A 70-year-old man presented with a conjunctival lesion in his left eye that had appeared four months earlier. Visual acuity (VA) was 20/200 in both eyes. Slit-lamp examination showed a nasal limbal gelatinous mass with inferior corneal infiltration and cataract (Figure 1 (Fig. 1)). Intraocular pressure and fundoscopy were unremarkable. An excisional biopsy was performed: A CIN with free margins was confirmed by the histopathological study (Figure 2 (Fig. 2)). Topical adjuvant treatment with interferon (IFN)-α2β drops/6 hours was initiated. One month later, the lesion decreased in size (Figure 3A (Fig. 3)), and topical treatment was reduced to 1 drop three times a day. At the third month, no evidence of CIN was observed (Figure 3B (Fig. 3)), and the patient has since remained free of disease.

### Case 2

**Figure 4:**
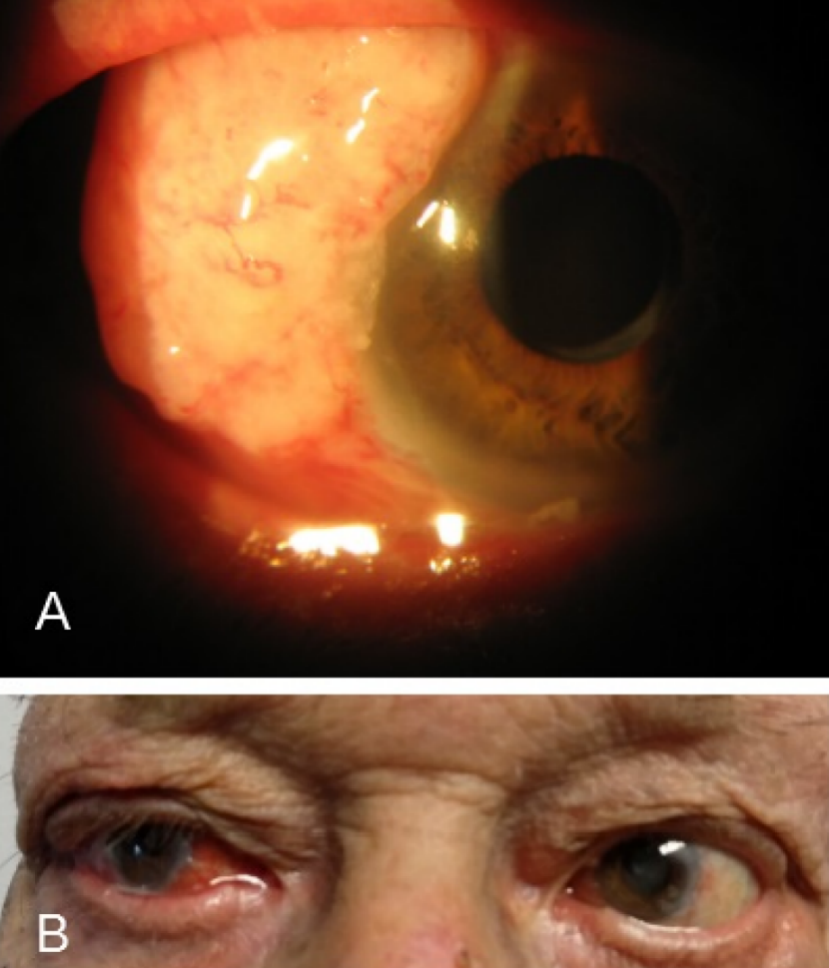
A) Slit-lamp examination: a gelatinous temporal conjunctival mass (7x10 mm) with dilated superficial vessels without corneal involvement is exposed. B) Abduction limitation in right eye.

**Figure 5:**
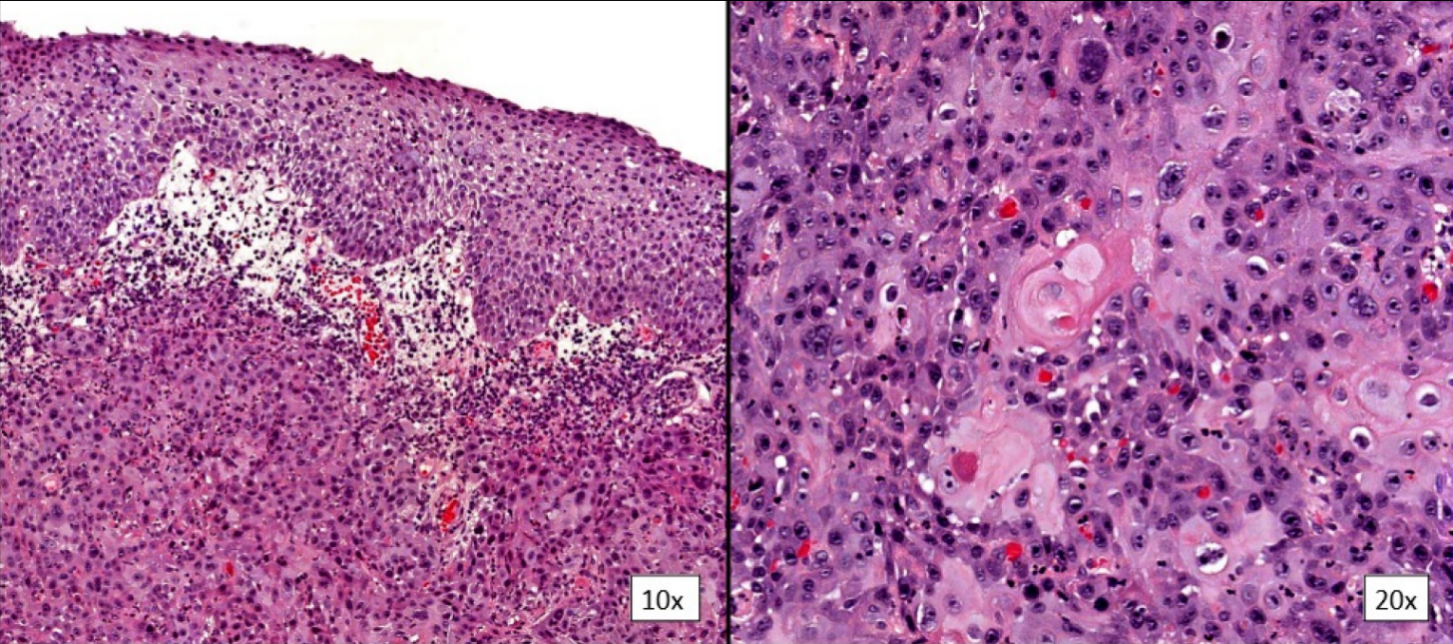
Anatomopathological study: moderately differentiated squamous cell carcinoma was confirmed; tumour cells present in corion

**Figure 6:**
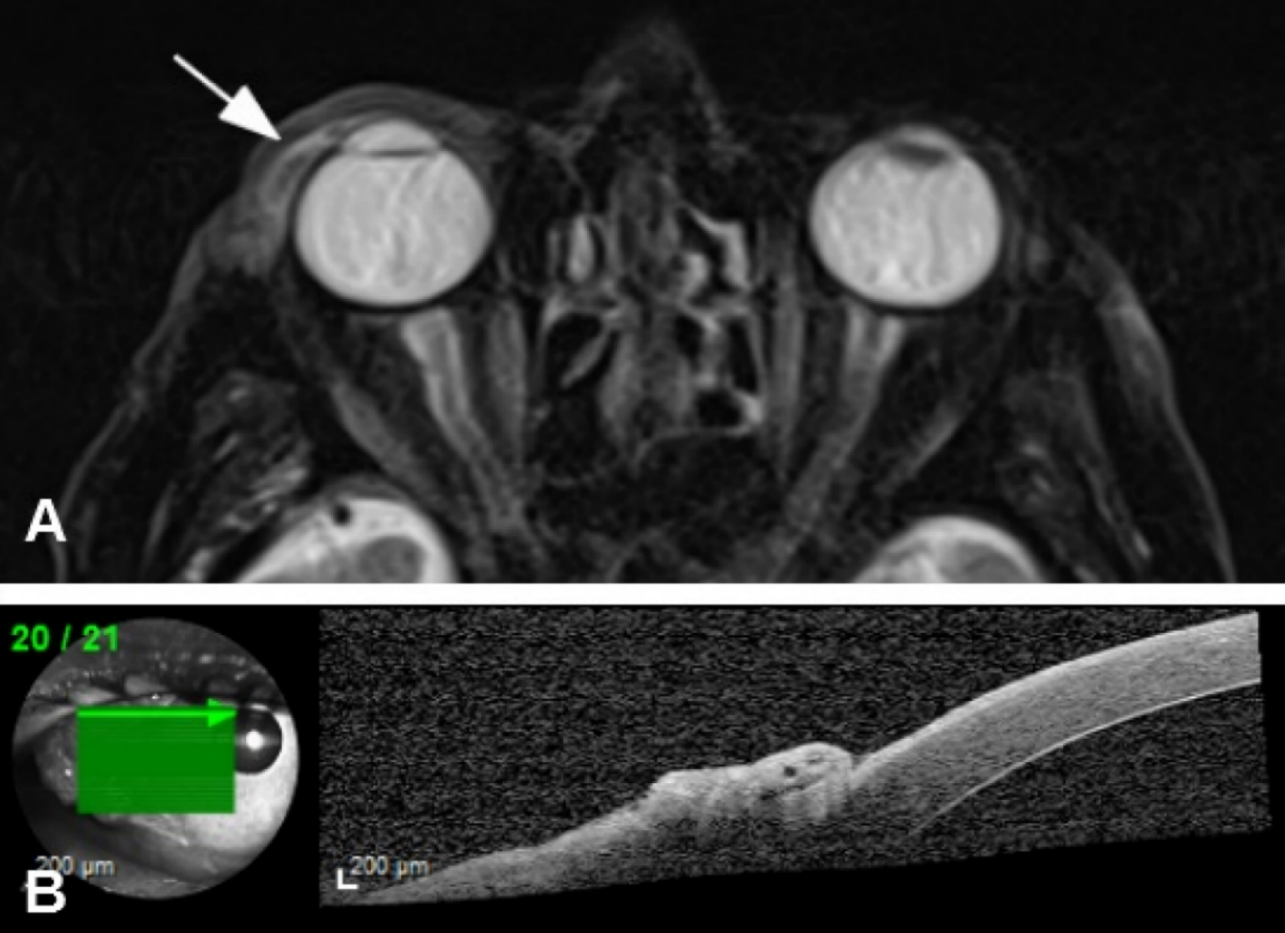
A) Magnetic Resonance Imaging showed lateral rectus muscle involvement. B) Anterior Segment – Optical Coherence Tomography (AS-OCT): a hyperreflective lesion involving conjunctival tissue and spread over corneal surface

**Figure 7:**
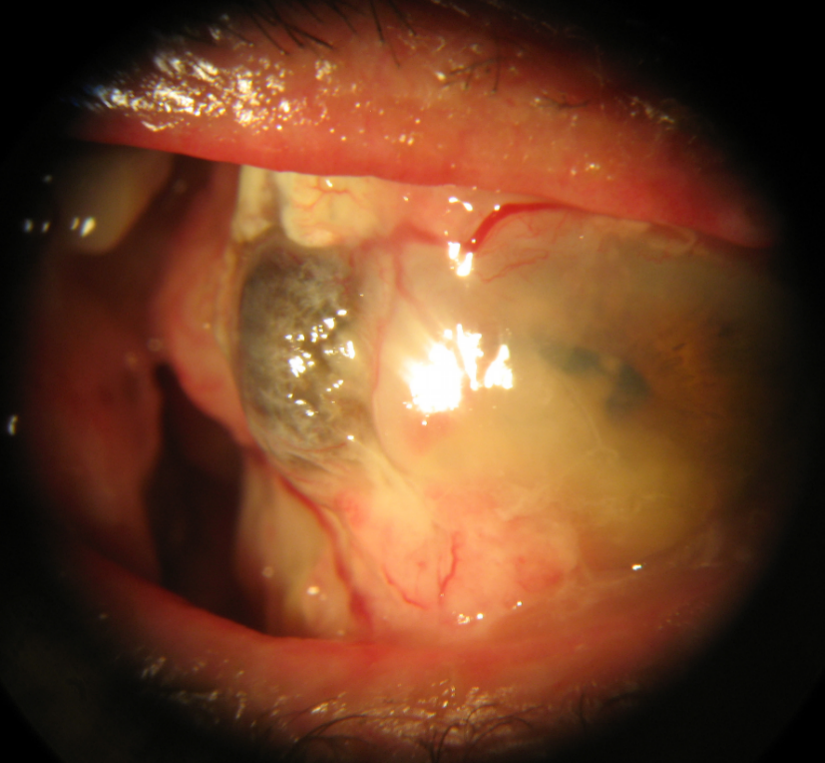
Tumor progression involving ocular globe tissues and soft periorbital structures

A 90-year-old man was referred to us with a mass in his right eye that had first been noticed three months earlier and had been growing progressively since then. VA was 20/200 in the right eye and hand movement in the left eye. Slit-lamp examination revealed a gelatinous temporal conjunctival mass (7 mm long, 10 mm wide) with dilated superficial vessels (Figure 4A (Fig. 4)). Fundus examination showed age-related macular degeneration (AMD) in both eyes. Ocular movements were affected in the form of abduction limitation in the right eye (Figure 4B (Fig. 4)). An incisional biopsy was performed and a moderately differentiated SCC was confirmed (Figure 5 (Fig. 5)). Magnetic resonance imaging (MRI) showed malignant infiltration of the lateral rectus muscle (Figure 6A (Fig. 6)). Anterior segment optical coherence tomography (AS-OCT) displayed a hyperreflective lesion which involved the conjunctival tissue and spread over the corneal surface (Figure 6B). Due to the advanced stage of the tumor, an exenteration or radiotherapy treatment was offered, but the patient rejected both. After five months, the tumor had progressed and involved ocular globe tissues and soft periorbital structures (Figure 7 (Fig. 7)). The patient died due to metastatic disease nine months after the initial diagnosis.

## Discussion

The incidence of epithelial conjunctival tumors is increasing due to higher ultraviolet light exposure, increased human papillomavirus infection rate [1], [4], and the rise in life expectancy. Other risk factors include a weakened immune system and tobacco exposure [5].

Epithelial conjunctival tumors are classified into one of two categories, depending on the degree to which the epithelial basement membrane is affected: CIN only affect the epithelium, while SCC spread to the basement membrane [1], [3]. The classification for conjunctival squamous neoplasia has been reviewed by the American Joint Committee on Cancer (AJCC) and is based on the depth of invasion as well as the size and extent of the affected adjacent structures [3].

CIN most commonly occurs in individuals in their 6^th^ or 7^th^ decade of life, and is divided into three dysplasia types: mild, moderate, and severe. It usually affects the interpalpebral fissure area in the form of a gelatinous, sessile, or papillomatous mass with irregular margins, which can spread over the corneal epithelium [1]. It is considered as a premalignant lesion and has a low risk of metastasizing. However, without rapid diagnosis and the establishment of appropriate treatment, there is a risk that atypical cells will invade the epithelial basement membrane and that the tumor will become a more malignant lesion with a worse prognosis.

Treatment is based on surgical excision with clear margins in combination with cryotherapy of the excised conjunctival edge. Cases with corneal involvement might also be managed with an alcohol epitheliectomy, which is in fact recommended [4]. Due to the irregular and unclear margins of the tumor, its correct excisional surgery can in some cases be a challenge for the surgeon. Recurrence rates range from 5 to 53% [1], [4], [6]. Adjuvant treatments with topical mitomycin C (MMC), 5-fluorouracil, or IFN-α2β are commonly used in order to avoid surgical procedures [4]. Excellent outcomes have been reported with topical IFN-α2β as primary treatment for conjunctival and/or corneal disease. It also plays a role as adjuvant therapy in cases with positive margins or recurrence after surgical excision [3]. As there is no established treatment protocol in terms of the duration over which IFN-α2β is administered, treatment must be individualized [2].

One third of patients develop irritative symptoms as side effects of surgery and chemotherapy, which often resolve quickly. One third of patients show radiation-induced complications that persist in the long term and consist of dry eye syndrome, lid alopecia, rubeosis, and secondary glaucoma [4].

The finding of epidermal growth factor receptor (EGFR) overexpression was an important aspect when considering targeted therapies in cases of inoperable advanced orbit and periocular SCC. EGFR inhibitors such as erlotinib have shown the effect of a significant decrease of SCC tumor size. Targeted therapy might be appropriate for non-surgical candidates, comprising patients with advanced metastatic orbital disease involving regional lymph nodes, advanced age, and/or patients with multiple comorbidities. This therapy is associated with fewer side effects and is better tolerated by patients [7].

Patients who reject treatment have a high mortality risk: it has been shown that these patients die after 13 months due to cancer progression [2]. The patient presented in case 2 chose to reject any treatment, resulting in cancer progression and death after 9 months.

All patients must be checked every 3–6 months because most recurrences occur during the first year of follow-up, and 30% of recurrences occur more than one year after surgical treatment.

Miller et al. recommended that advanced stage SCC should be closely monitored as recurrences can occur even more than 5 years after treatment [2].

## Conclusion

Early diagnosis is essential in epithelial conjunctival tumors. Histopathological examination allows a classification of the lesions as premalignant or malignant, which is crucial for the development of a therapeutic strategy.

An excisional biopsy with cryotherapy and free margins is sufficient in most cases of CIN. Topical chemotherapy can be used additionally. In cases of SCC, surgical excision may be sufficient, but may also be combined with adjuvant topical therapy, which may enhance treatment in cases of insufficient surgical removal. In addition, when SCC penetrates the intraocular or orbital septum, enucleation, exenteration, radiotherapy, or targeted therapy have to be performed on each specific case.

## Notes

### Competing interests

The authors declare that they have no competing interests.

### Informed consent

Informed consent has been obtained from the patient for the publication of this case report.
